# Ischemic perfusion radiomics: assessing neurological impairment in acute ischemic stroke

**DOI:** 10.3389/fneur.2024.1441055

**Published:** 2024-07-16

**Authors:** Jiaxi Lu, Mazen M. Yassin, Yingwei Guo, Yingjian Yang, Fengqiu Cao, Jiajing Fang, Asim Zaman, Haseeb Hassan, Xueqiang Zeng, Xiaoqiang Miao, Huihui Yang, Anbo Cao, Guangtao Huang, Taiyu Han, Yu Luo, Yan Kang

**Affiliations:** ^1^School of Applied Technology, Shenzhen University, Shenzhen, China; ^2^College of Health Science and Environmental Engineering, Shenzhen Technology University, Shenzhen, China; ^3^School of Biomedical Engineering, Shenzhen University Medical School, Shenzhen University, Shenzhen, China; ^4^School of Electrical and Information Engineering, Northeast Petroleum University, Daqing, China; ^5^Department of Radiological Research and Development, Shenzhen Lanmage Medical Technology Co., Ltd., Shenzhen, China; ^6^School of Information Science and Engineering, Shenyang Polytechnic University, Shenyang, China; ^7^Shenzhen Academy of Metrology and Quality Inspection, Shenzhen, China; ^8^College of Medicine and Biological Information Engineering, Northeastern University, Shenyang, China; ^9^Department of Radiology, Shanghai Fourth People’s Hospital Affiliated to Tongji University School of Medicine, Shanghai, China; ^10^Engineering Research Centre of Medical Imaging and Intelligent Analysis, Ministry of Education, Shenyang, China

**Keywords:** neurological impairment, acute ischemic stroke, DSC-PWI, perfusion parameters, radiomics

## Abstract

**Introduction:**

Accurate neurological impairment assessment is crucial for the clinical treatment and prognosis of patients with acute ischemic stroke (AIS). However, the original perfusion parameters lack the deep information for characterizing neurological impairment, leading to difficulty in accurate assessment. Given the advantages of radiomics technology in feature representation, this technology should provide more information for characterizing neurological impairment. Therefore, with its rigorous methodology, this study offers practical implications for clinical diagnosis by exploring the role of ischemic perfusion radiomics features in assessing the degree of neurological impairment.

**Methods:**

This study employs a meticulous methodology, starting with generating perfusion parameter maps through Dynamic Susceptibility Contrast-Perfusion Weighted Imaging (DSC-PWI) and determining ischemic regions based on these maps and a set threshold. Radiomics features are then extracted from the ischemic regions, and the *t*-test and least absolute shrinkage and selection operator (Lasso) algorithms are used to select the relevant features. Finally, the selected radiomics features and machine learning techniques are used to assess the degree of neurological impairment in AIS patients.

**Results:**

The results show that the proposed method outperforms the original perfusion parameters, radiomics features of the infarct and hypoxic regions, and their combinations, achieving an accuracy of 0.926, sensitivity of 0.923, specificity of 0.929, PPV of 0.923, NPV of 0.929, and AUC of 0.923, respectively.

**Conclusion:**

The proposed method effectively assesses the degree of neurological impairment in AIS patients, providing an objective auxiliary assessment tool for clinical diagnosis.

## 1 Introduction

Acute ischemic stroke (AIS) is a prevalent neurological condition that imposes a significant health burden worldwide (1). With the advancements in imaging technology and medical interventions, stroke mortality rates have declined. Nevertheless, the neurological impairment caused by stroke leads to varying degrees of disability and severely impacts patients’ quality of life (2). Furthermore, the degree of neurological impairment plays a pivotal role in clinical management and treatment choices for AIS patients (3). Thus, precise assessing of neurological impairment has immense importance in stroke recovery prognosis and treatment strategies.

The National Institutes of Health Stroke Scale (NIHSS) is the most widely used tool to assess neurological impairments in clinical practices (4). It comprehensively assesses the patients in 11 dimensions, including consciousness, vision, motor function, sensation, and language, etc., classifying them into four severity levels. A higher score represents severe damage, and further research has shown a correlation between NIHSS and the prognosis of stroke patients with post-treatment NIHSS evaluations, which are commonly employed for assessing short-term functional recovery (5, 6). However, the NIHSS entirely relies on clinical manifestations, which lack direct insight into brain tissue pathology and physiological processes. This limitation is prone to subjectivity and certain constraints. Particularly reliability issues such as facial paralysis, ataxia, and level of consciousness. Differences in how different accessors interpret scoring criteria may result in inconsistent scores for the same patient, and some patients with an NIHSS score of 0 may still have experienced a stroke (7).

Cerebral hemodynamic parameters are essential in characterizing brain tissue’s blood supply and transportation capacity (8). When there is an ischemic stroke event, blood vessel blockages occur, which leads to restricted cerebral blood flow. As a result, tissues in the affected region sustain varying degrees of damage due to oxygen deprivation. Diffusion-weighted imaging (DWI) is a method that reflects the restricted diffusion of extracellular water molecules. If there is a decrease in the apparent diffusion coefficient (ADC), it indicates the early changes in brain infarction (9).

Although DWI is commonly used for early diagnosis of ischemic stroke, it cannot reflect the blood supply to the brain. Dynamic susceptibility contrast-perfusion weighted imaging (DSC-PWI) is valuable for microvascular regeneration and blood perfusion status. Moreover, it provides perfusion parameters such as cerebral blood flow (CBF), cerebral blood volume (CBV), mean transient time (MTT), time to maximum of the residual function (Tmax), and more. Thus, DSC-PWI has emerged as the predominant imaging technique in current research focused on blood perfusion assessment (10), and several studies have investigated the association between perfusion parameters and stroke. Aracki et al. (11) found that the ischemic regions’ absolute CBF and relative CBF in AIS patients correlated with functional prognosis. The area under the curve (AUC) reached 0.914 and 0.842, respectively. Aicheng et al. (12) observed a significant association between the CBV index of the ischemic core and functional outcomes (OR, 0.001; 95% CI 0.000–0.240, *p* = 0.014). Shin et al. (13) identified a close association between post-thrombectomy time to peak (TTP) hypoperfused volume and TTP hypoperfused volume change early neurological improvement. The AUC reached 0.90 and 0.82, respectively. In summary, it is evident that quantitative perfusion parameters directly reflect the blood perfusion of brain tissue, which is conducive to patients’ status assessment and prognosis prediction. However, this evaluation method is solely based on perfusion parameters and neglects the deeper information within perfusion parameter maps. This limitation makes it difficult to comprehensively analyze the blood flow transmission process and the degree of tissue damage. Consequently, it hinders in-depth research on AIS disease’s occurrence, development, and subsequent recovery mechanisms.

Cerebral oxygen metabolism levels indicate the neurological functional vitality of brain tissue, with changes typically closely related to the brain’s functional state (14). Quantitative sensitivity mapping (QSM) is an emerging magnetic resonance (MR) imaging technique that has been demonstrated to non-invasively reflect oxygen extraction fraction (OEF) and cerebral venous oxygen saturation (SvO_2_) (15). In a prospective study of large vessel occlusion type AIS, Uchida et al. (16) discovered that volumes exhibiting increased OEF values surpassing 51.5% were positively correlated with the ischemic penumbra volumes (*r* = 0.636, 95% CI: 0.059 to 0.895, *p* = 0.035). Additionally, they found an inverse correlation between these volumes and the 30 day change in the National Institutes of Health Stroke Scale (NIHSS) scores (*r* = −0.624, 95% CI: −0.891 to −0.039, *p* = 0.041). In another work, Xia et al. (17) measured the susceptibility of cortical veins in the stroke and contralateral hemispheres using QSM. They found that the oxygen saturation in the asymmetrically prominent cortical veins (APCV) was reduced by 16–44% compared to the contralateral hemisphere.

Cerebral SvO_2_ reflects the degree of oxygen saturation in the cerebral venous system, an essential index for assessing brain tissue’s oxygenation level. However, there is limited research on quantifying SvO_2_ using QSM and a lack of unified and accurate quantification standards (18). Therefore, in the comparative experiment of this study, SvO_2_ calculation is considered and generated SvO_2_ maps which are used to determine the location of cerebral hypoxic regions. This research aims to investigate the significance and utility of perfusion radiomics features within these hypoxic regions to assess the degree of neurological impairment rather than establish a direct correlation between SvO_2_ and neurological impairment.

In summary, cerebral hemodynamic parameters and cerebral oxygen metabolism levels are correlated with the degree of neurological impairment to a certain extent. However, further exploration is warranted due to the limited characterization ability of the original parameters and the inherent advantages of radiomics features in delineating the degree of neurological impairment. This paper proposes a method for assessing the degree of neurological impairment in AIS patients based on radiomics features of the ischemic regions in perfusion parameter maps, aiming to provide an accurate and objective assessment tool for neurological impairment and improve patients’ treatment and rehabilitation outcomes. Additionally, multiple comparative experiments were conducted to comprehensively analyze the role of radiomics features and corresponding original perfusion parameters extracted from the ischemic, infarct, and hypoxic regions of perfusion parameter maps in assessing the degree of neurological impairment in AIS patients.

## 2 Materials

**Figure 1:**
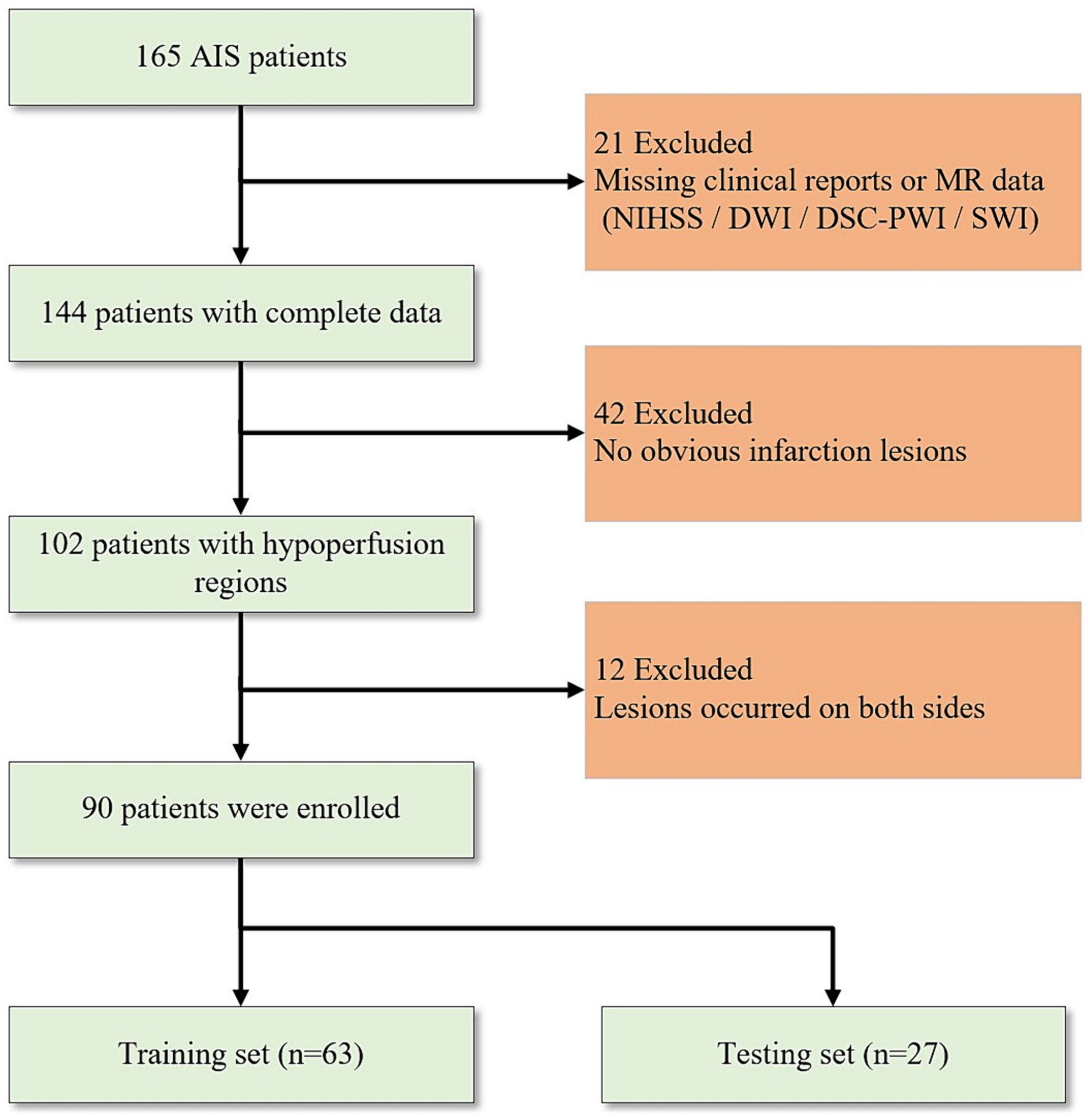
Flowchart of patient inclusion and exclusion criteria.

The study conducted a retrospective analysis of 165 AIS patients hospitalized in the neurology department of the Shanghai Fourth People’s Hospital, affiliated with the Tongji University School of Medicine, China, from June 2013 to July 2019. The institutional review boards agreed on this retrospective study and exempted it from informed consent. The inclusion criteria are based on the following: (1) patients underwent MR scanning within 24 h after symptom onset; (2) Complete MR sequences of DWI, DSC-PWI, and susceptibility weighted imaging (SWI); (3) Comprehensive clinical reports and NIHSS scores; (4) presence of evident infarct lesions observable on DWI; and (5) occurrence of unilateral lesions with the contralateral hemisphere remaining unaffected. Finally, a total number of 90 AIS patients were considered (Figure 1). Despite certain limitations, NIHSS remains the preferred clinical measure to access the neurological impairment. Thus, we still considered the NIHSS, obtained upon patient admission, as it is the gold standard for this research. Selected patients were categorized into two groups based on their NIHSS scores. Those with good neurological function (NIHSS: 0–4) and those with poor neurological function (NIHSS: 5–42). Further, the patients were divided into training and testing sets at a ratio of 7:3. All MR imaging data were acquired using a 1.5-Tesla MR scanner (Siemens, Erlangen, Germany). Table 1 presents the summary of sequence parameters.

**Table 1:** Summary of sequence parameters.

Parameters	DSC-PWI	DWI	SWI
Matrix size	256 × 256	192 × 192	260 × 320
Slices	20	20	72
Slice thickness (mm)	5	5	1.6
Pixel spacing (mm × mm)	0.898 × 0.898	1.198 × 1.198	0.718 × 0.718
TR (ms)	1,520	3,600	49
TE (ms)	32	102	40
FOV (mm × mm)	230 × 230	230 × 230	230 × 230
Bandwidth (Hz/pixel)	1,347	965	80
measurements	50	/	/

## 3 Methods

### 3.1 Perfusion parameter calculation and ischemic regions segmentation

**Figure 2:**
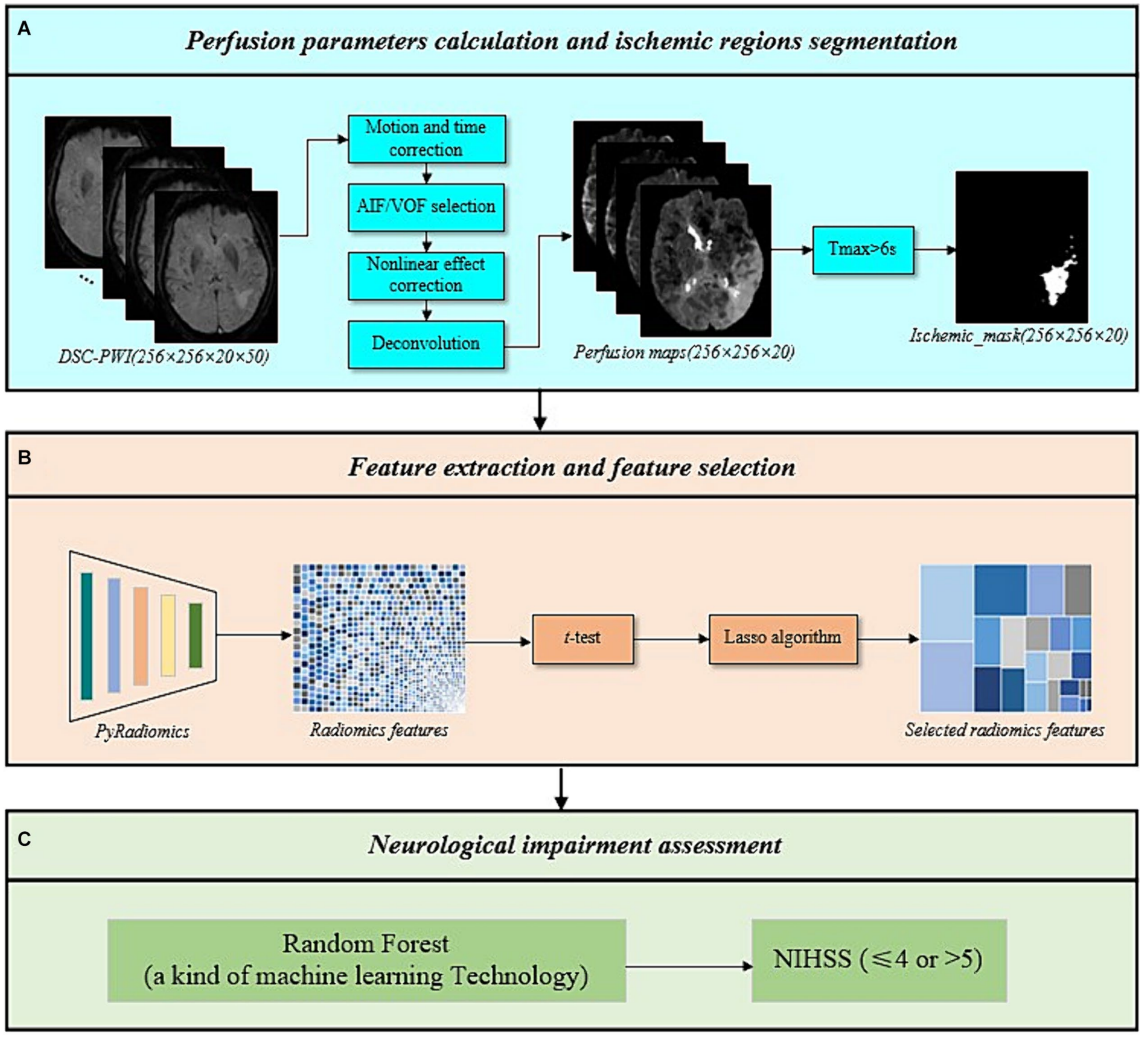
The overall framework of the proposed method. **(A)** Perfusion parameters calculation and ischemic regions segmentation. **(B)** Feature extraction and feature selection. **(C)** Neurological impairment assessment.

This study utilized DSC-PWI to calculate cerebral perfusion parameters quantitatively (19). Initially, motion correction and time adjustments were performed on the DSC-PWI, converting the MR signal to estimated changes of transverse relaxivity. Subsequently, automatic detection of arterial input function (AIF) and venous output function (VOF) was conducted, followed by correction for the nonlinear effects of gadolinium tracer in blood and capillaries. Finally, perfusion parameters were calculated using deconvolution and generated perfusion parameter maps. Ischemic regions were delineated using the threshold of Tmax >6 s. As shown in Figure 2A. This calculation was conducted using RAPID software (v2017; iSchemaView, Menlo Park, CA, United States). The conducted perfusion parameters include CBF, CBV, MTT, and Tmax.

### 3.2 Feature extraction and feature selection

Following the above process, four perfusion parameter maps (CBF, CBV, MTT, Tmax) and ischemic masks were obtained. This study extracted radiomics features from the ischemic regions of the four perfusion parameter maps using radiomics techniques. The radiomics techniques used included six categories of original features: first order, gray level co-occurrence matrix (GLCM), gray level size zone matrix (GLSZM), gray level run length matrix (GLRLM), neighboring gray-tone difference matrix (NGTDM), and gray level dependence matrix (GLDM) (20, 21). To capture feature information across different frequency domains and under various deformations, six filters were applied to process the original features, including log sigma with scale {1.0, 2.0, 3.0, 4.0, 5.0}, wavelet, square, square root, logarithm, and exponential.

Subsequently, a feature selection was conducted on the extracted radiomics features. Prior to feature selection, feature vectors were standardized using Eq. 1 to eliminate the influence of differences in dimensionality and value between features and to expedite computation. Next, a *t*-test was conducted on the standardized features to retain those with significant values (*p*-value <0.05), reducing the dimensionality. Afterward, the least absolute shrinkage and selection operator (Lasso) algorithm was applied to eliminate variables with smaller parameter estimates, further simplifying the feature set (22). Only features with non-zero weights were kept. Finally, features from the ischemic regions of the four perfusion parameter maps were combined to create the final set of ischemic perfusions radiomics features, as shown in Figure 2B. The Lasso algorithm was implemented using the LassoCV function in scikit-learn.


(1) $$F_{i}^{*}=\frac{F_{i}-\bar{F_{t}}}{F_{\mathrm{imax}}-F_{\mathrm{imin}}}$$

where $F_{i}^{*}$ is the standardized result of the feature $F_{i}$ , the variables $\bar{F_{t}}$ , $F_{\mathrm{imax}}$ and $F_{\mathrm{imin}}$ are the mean, maximum, and minimum of $F_{i}$ , respectively, and the *i* is the order of features.

### 3.3 Neurological impairment assessment

To evaluate the performance of ischemic perfusion radiomics features in assessing the neurological impairment in AIS patients, six machine learning classifiers were constructed using the scikit-learn, including Support Vector Machine (SVM), Multi-Layer Perceptron (MLP), Random Forest (RF), Adaboost Classifier (Ada), Logistic Regression (LR), and Gaussian Naive Bayes (NB) (depicted in Figure 2C). Validation was performed on an independent testing set to evaluate accuracy, sensitivity, specificity, positive predictive value (PPV), negative predictive value (NPV), and AUC.

### 3.4 Comparative experimental design

**Figure 3:**
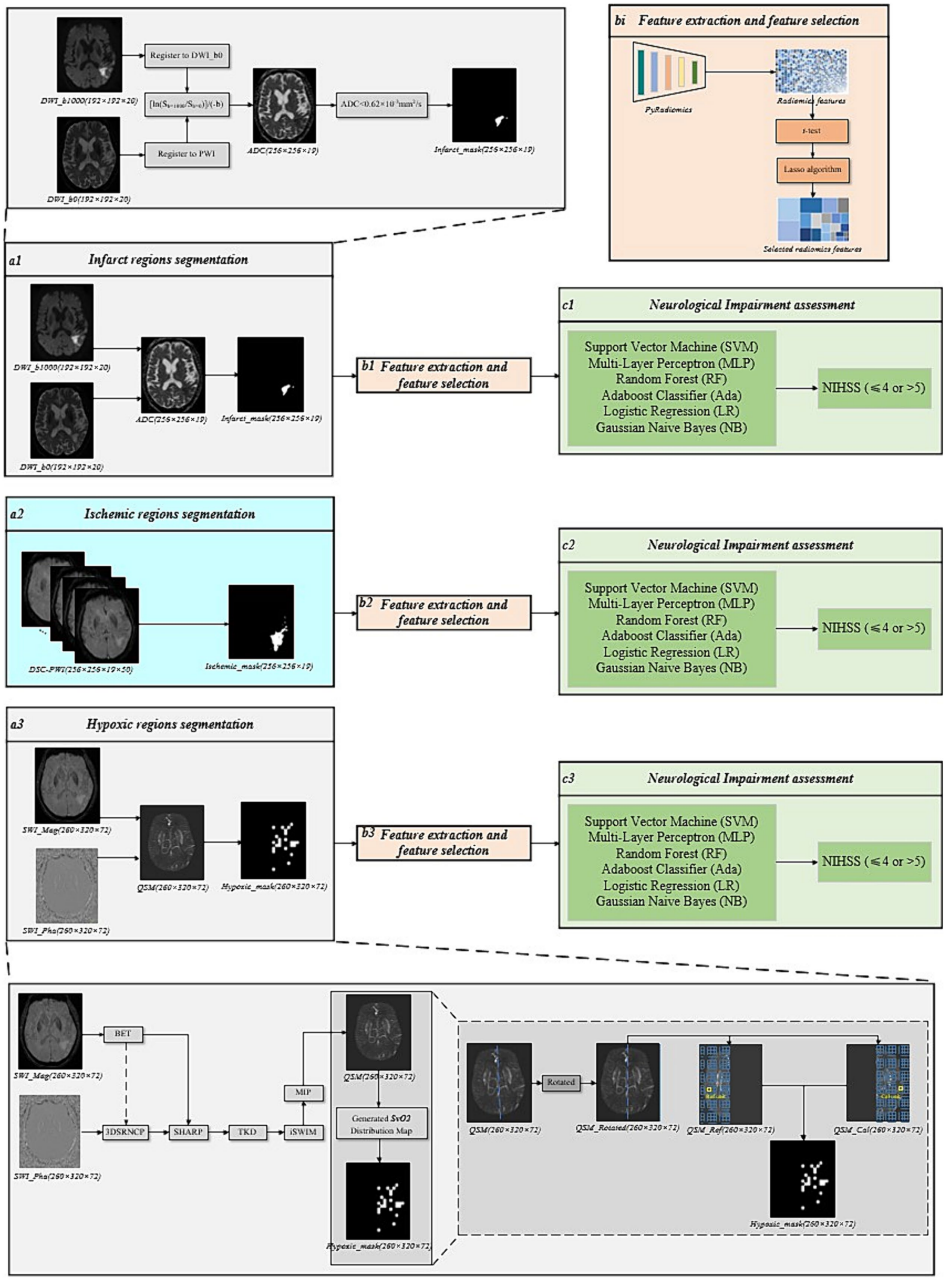
The overall framework of the comparative experimental groups. **(a1)** Infarct regions segmentation. **(a2)** Ischemic regions segmentation. **(a3)** Hypoxic regions segmentation. **(bi)** Feature extraction and feature selection. **(ci)** Neurological impairment assessment.

This study designed multiple sets of comparative experiments to comprehensively analyze the role of radiomics features and corresponding original perfusion parameters extracted from ischemic, infarct, and hypoxic regions of perfusion parameter maps in assessing the degree of neurological impairment in AIS patients. Firstly, infarct and hypoxic regions were delineated based on DWI and QSM, respectively, and radiomics features were extracted from these regions’ four perfusion parameter maps using the methods above. After feature selection and combination, the final infarct perfusion radiomics features and hypoxic perfusion radiomics features were obtained. Finally, six machine learning classifiers were used to evaluate the performance of the perfusion radiomics features in each region. The overall process is provided in Figure 3. Simultaneously, the original perfusion parameters (CBF, CBV, MTT, and Tmax) were calculated for the ischemic, infarct, and hypoxic regions. These four parameters were then combined according to their respective regions to obtain the final ischemic, infarct, and hypoxic original perfusion parameters. Statistical analyses were performed using IBM SPSS Statistics, version 26.0 (IBM Corp., Armonk, NY, United States), and independent sample *t*-test was used to compare the statistical differences of continuous variables. The aforementioned machine learning classifiers were used to evaluate the performance of original perfusion parameters in the ischemic, infarct, and hypoxic regions, aiming to compare the performance of perfusion radiomics features and original perfusion parameters.

Furthermore, to determine if combining features and parameters from different regions could enhance evaluation performance, the radiomics features, and perfusion parameters from the ischemic, infarct, and hypoxic regions were separately combined. These combinations were renamed as experimental groups using the format “region_radiomics/parameters.” For example, “Ischemic_infarct_radiomics” represents the experimental group that combined perfusion radiomics features of ischemic and infarct regions, whereas “Ischemic_hypoxic_parameters” refers to the experimental group that combined perfusion parameters of both ischemic and hypoxic regions.

#### 3.4.1 Infarct regions segmentation

The segmentation of infarct regions was performed based on DWI. Firstly, the DWI images with *b* = 0 s/mm^2^ and *b* = 1,000 s/mm^2^ were registered to the DSC-PWI, and the ADC values were calculated based on Eq. 2 (19). Finally, an absolute ADC threshold (ADC < 620 × 10^−6^ mm^2^/s) was set to delineate the infarct regions. This process was performed using the RAPID software, as shown in Figure 3a1.


(2) $$ADC=-\frac{1}{b}\ln\left(\frac{S_{b=1000}}{S_{b=0}}\right)$$

where *b* is the diffusion weighting factor, $S_{b=0}$ is the signal value when diffusion gradients are turned off.

#### 3.4.2 Hypoxic regions segmentation

For the segmentation of the Hypoxic region, the Hypoxic regions were delineated based on the calculation of SvO_2_ from QSM images. However, prior to calculating the SvO_2_, the QSM needed to be reconstructed. The reconstruction of QSM involved several post-processing steps on the phase and amplitude images of SWI. This procedure includes phase unwrapping, background field contributions, susceptibility inversion algorithm, and maximum intensity projection (MIP) (23), as illustrated in Figure 3a3. The brain tissue masks were obtained from amplitude images using the brain extraction tool (BET) (24). The sophisticated harmonic artifact reduction for phase (SHARP) technique was used with a kernel size of 8 and a deconvolution threshold of 0.05 to eliminate background field contributions (25). To further enhance the visualization of the veins and deep gray matter tissues and to remove stripe artifacts, the reconstructed QSM underwent an iterative SWIM (iSWIM) algorithm with four iterations and a 16-layer MIP (26).

Considering the susceptibility of draining veins in the affected brain tissue units in QSM, the SvO_2_ parameters were calculated. To segment the QSM to extract the susceptibility values of aberrant draining veins, a lower threshold of 90 ppb was set, while to remove points with exceptionally high values, a higher threshold of 300 ppb was set. Further, to ensure the brain remained upright and divided into left and right hemispheres along the brain’s midline, the QSM was rotated based on the centroid and deviation angle. According to previous studies, a grid division of 10 × 10 × 1 produced SvO_2_ results most predictive of prognostic outcomes (27). Consequently, in our experiments, we utilized this grid size to partition calculation and reference units for the affected and healthy hemispheres, as illustrated in Figure 3a3. The mathematical relationship between cerebral veins susceptibility and SvO_2_ is represented by Eq. 3.


(3) $$\Delta X_{\mathrm{vein}-\mathrm{tissue}}=\Delta_{x_{\mathrm{do}}}\times \left(1-{SvO}_{2}\right)\times Hct$$

where $\Delta X_{\mathrm{vein}-\mathrm{tissue}}$ represents the susceptibility difference between veins and surrounding brain tissues (28), and the susceptibility difference between totally oxygenated blood and deoxygenated blood is represented by $\Delta_{x_{\mathrm{do}}}$ = 0.18 ppm (29), whereas Hct is the hematocrit of large, draining veins (30). Assuming the susceptibility of brain tissues without draining veins is 0 ( $\Delta X_{\mathrm{vein}-\mathrm{tissue}}=X_{\mathrm{vein}}$ ) (31). Simultaneously, to eliminate uncertain constants of Hct and $\Delta_{x_{\mathrm{do}}}$ , the change of cerebral SvO_2_ ( $\Delta {{SvO}_{2}}_{\left(ROI\right)}$ ) is introduced.


(4) $$\Delta {{SvO}_{2}}_{\left(ROI\right)}={{SvO}_{2}}_{\left(Ref\right)}-{{SvO}_{2}}_{\left(ROI\right)}$$

where ${{SvO}_{2}}_{\left(Ref\right)}$ is the SvO_2_ of the reference unit, ${{SvO}_{2}}_{\left(ROI\right)}$ is the SvO_2_ of the calculation unit. Finally, the ratio of $\Delta {{SvO}_{2}}_{\left(ROI\right)}$ to ${{SvO}_{2}}_{\left(ROI\right)}$ is calculated, and the simplified formula is obtained.


(5) $$\Delta {{SvO}_{2}}_{\left(ROI\right)}=-\left(1-{{SvO}_{2}}_{\left(Ref\right)}\right)\times \frac{X_{\mathrm{vein}\left(Ref\right)}-X_{\mathrm{vein}\left(ROI\right)}}{X_{\mathrm{vein}\left(Ref\right)}}$$

where $X_{\mathrm{vein}\left(Ref\right)}$ is the susceptibility of draining veins of the reference unit, $X_{\mathrm{vein}\left(ROI\right)}$ is the susceptibility of draining veins of the calculation unit. We assume the SvO_2_ to be within the normal range ( ${{SvO}_{2}}_{\left(Ref\right)}=0.7$ ) for the contralateral hemisphere (32).

The SvO_2_ of the calculation units can be calculated by measuring the SvO_2_ of the reference units and changes of cerebral SvO_2_. Calculation units with SvO_2_ lower than 0.7 are defined as hypoxic regions. Additionally, image registration is required due to the discrepancies between QSM and perfusion parameter maps. For this purpose, initially, FMRIB’s Linear Image Registration Tool (FLIRT) a neuroimaging software package was employed to register the first-time-point DSC-PWI to the SWI through a 12-degree-of-freedom affine transformation resulting in conversion matrix (33, 34). Subsequently, the perfusion parameter maps were registered to the SWI based on the conversion matrix.

## 4 Results

### 4.1 Patient characteristics

A total of 90 AIS patients were included in the study, consisting of 65 males and 25 females, with an age range from 45 to 93 years (Table 2). There were 45 patients with good neurological function, with an average age of 70.6 ± 10.5 years, and average NIHSS score of 1.9 ± 1.3. There were 45 patients with poor neurological function, with an average age of 70.2 ± 10.6 years, and average NIHSS score of 12.4 ± 5.7. Of the 90 enrolled patients, the training set included 63 cases, with 31 of good neurological function and 32 of poor neurological function. The testing set comprises 27 cases, which have 14 of good neurological function and 13 of poor neurological function.

**Table 2:** Patient information with good and poor neurological function.

Characteristics	Good neurological function (*n* = 45)	Poor neurological function (*n* = 45)
Age (years)	70.6 ± 10.5	70.2 ± 10.6
Gender(male/female)	30/15	35/10
Hypertension	37	37
Diabetes	12	15
Atrial fibrillation	12	14
Lisp out	28	29
Infarction side (R/L)	24/21	22/23
NIHSS	1.9 ± 1.3	12.4 ± 5.7

### 4.2 Perfusion parameter maps and masks

**Figure 4:**
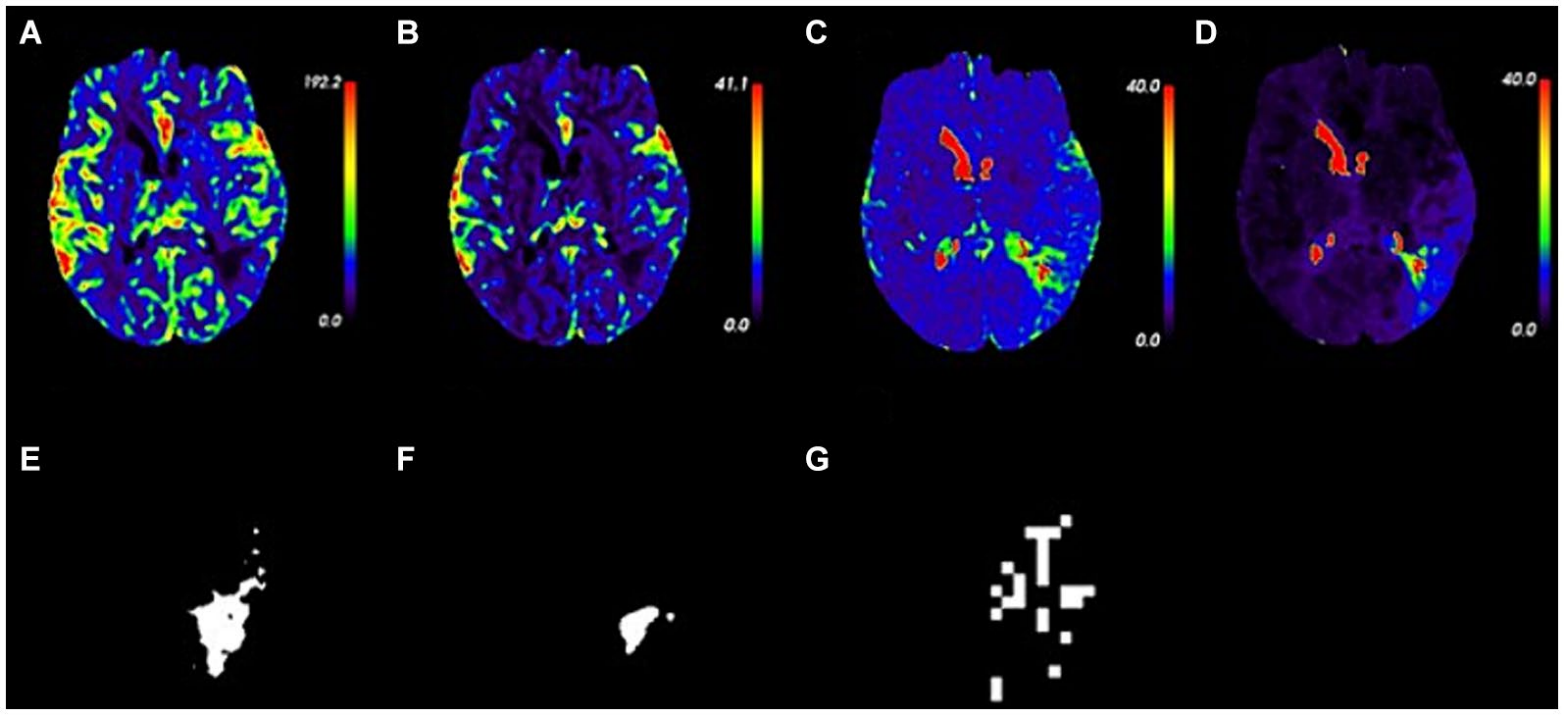
Perfusion parameter maps and masks in an 81 year-old patient with acute ischemic stroke. **(A–D)** Perfusion parameter maps of CBF, CBV, MTT, and Tmax. **(E–G)** Masks of ischemic, infarct, and hypoxic regions.

Perfusion parameter maps (CBF, CBV, MTT, and Tmax) and masks of ischemic, infarct, and hypoxic regions for an 81-year-old male AIS patient are illustrated in Figure 4. Five hours after symptom onset, the patient exhibited weakness in the left limbs and mild dysarthria, resulting in an NIHSS score of 2. Imaging showed lesions in the left hemisphere of the brain.

### 4.3 Selected perfusion radiomics features

**Figure 5:**
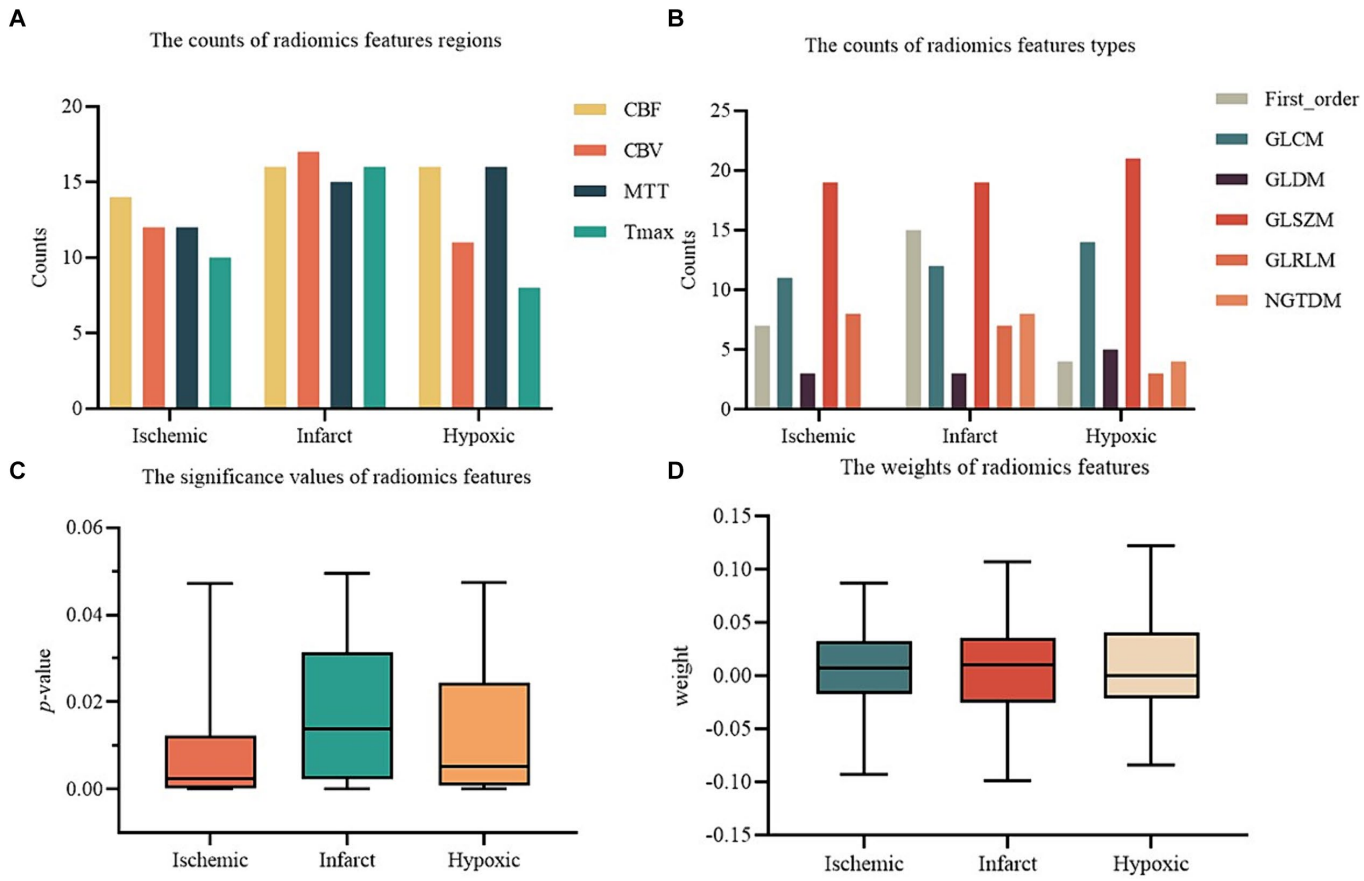
The results of radiomics features selection. **(A)** The counts of radiomics features regions. **(B)** The counts of radiomics features types. **(C)** The significance values of radiomics features. **(D)** The weights of radiomics features.

The results of radiomics feature selection for ischemic, infarct, and hypoxic regions in perfusion parameter maps are shown in Figure 5. For the ischemic region, a total of 48 radiomics features were retained, comprising 14 from CBF, 12 from CBV, 12 from MTT, and 10 from Tmax maps. These features have different types: 7 in First order, 11 in GLCM, 3 in GLDM, 19 in GLSZM, 8 in GLRLM, and none from NGTDM. The mean *p*-value of features was 0.008 ± 0.011, with a feature weight of 0.007 ± 0.042. For the infarct region, 64 radiomics features were retained, including 16 from CBF, 17 from CBV, 15 from MTT, and 16 from Tmax maps. A total of 15 features were included in the First order, 12 in GLCM, 3 in GLDM, 19 in GLSZM, 7 in GLRLM, and 8 in NGTDM. The mean *p*-value was 0.018 ± 0.016, and the feature weight was 0.007 ± 0.047. In the hypoxic region, 51 radiomics features were retained, comprising 16 from CBF, 11 from CBV, 16 from MTT, and 8 from Tmax maps. Four features were kept for the First order, 14 in GLCM, 5 in GLDM, 21 in GLSZM, 3 in GLRLM, and 4 in NGTDM. The mean *p*-value was 0.013 ± 0.014, and the feature weight was 0.007 ± 0.042.

### 4.4 Performance of ischemic perfusion radiomics features

This study evaluated the classification performance of machine learning models on the NIHSS of AIS patients to analyze the performance of different feature combinations in assessing the degree of neurological impairment. Table 3 shows the performance of radiomics features extracted from ischemic regions of perfusion parameter maps after feature selection. This feature combination demonstrated the best performance among all experimental groups and was the recommended feature combination in this study. The RF model exhibited the best overall performance, with an accuracy of 0.926, sensitivity of 0.923, specificity of 0.929, PPV of 0.923, NPV of 0.929, and AUC of 0.923, respectively.

**Table 3:** Performance of perfusion radiomics features in ischemic regions.

Classifier	Accuracy	Sensitivity	Specificity	PPV	NPV	AUC
SVM	0.852	0.846	0.857	0.846	0.857	0.945
MLP	0.815	0.923	0.714	0.750	0.909	0.962
RF	0.926	0.923	0.929	0.923	0.929	0.923
Ada	0.741	0.769	0.714	0.714	0.769	0.808
LR	0.815	0.923	0.714	0.750	0.909	0.951
NB	0.852	0.923	0.786	0.800	0.917	0.923

### 4.5 Original perfusion parameters

Table 4 shows the original perfusion parameters of ischemic, infarct, and hypoxic regions in 90 AIS patients and summarizes the differences between patients with good and poor neurological function. All other perfusion parameters showed significant differences except for the CBV parameter in the ischemic region. Specifically, CBF, MTT, and Tmax parameters exhibited highly significant differences (*p*-value <0.01). Patients with good neurological function had significantly higher CBF than those with poor neurological function. In contrast, MTT and Tmax were significantly lower in patients with good neurological function.

**Table 4:** The original perfusion parameters in ischemic, infarct, and hypoxic regions.

Perfusion parameters	Good neurological function (*n* = 45)	Poor neurological function (*n* = 45)	*p*-value
Ischemic			
CBF	28.05 ± 11.90	20.34 ± 9.47	0.001**
CBV	3.83 ± 1.44	3.49 ± 1.11	0.214
MTT	9.42 ± 3.27	13.67 ± 5.31	0.000**
Tmax	6.24 ± 4.49	12.18 ± 6.28	0.000**
Infarct			
CBF	28.22 ± 16.56	17.81 ± 10.81	0.001**
CBV	3.54 ± 1.59	2.88 ± 1.31	0.034*
MTT	9.53 ± 4.49	14.73 ± 5.83	0.000**
Tmax	5.80 ± 5.33	13.99 ± 8.39	0.000**
Hypoxic			
CBF	58.54 ± 18.50	47.36 ± 18.83	0.006**
CBV	7.91 ± 2.09	6.94 ± 2.11	0.031*
MTT	8.13 ± 1.19	9.51 ± 2.22	0.000**
Tmax	3.65 ± 0.97	5.32 ± 2.43	0.000**

*p-value <0.05 and **p-value <0.01.

### 4.6 Performance of comparative experimental groups

We evaluated perfusion radiomics features’ performance in infarct and hypoxic regions, as shown in Table 5. In the “Infarct_radiomics” group, the performance of the models was significantly worse than that of the ischemic regions, with the NB model exhibiting the best overall performance, achieving an accuracy of 0.741, sensitivity of 0.769, specificity of 0.714, PPV of 0.714, NPV of 0.769, and AUC of 0.821, respectively. The optimal model in the “Hypoxic_radiomics” group was LR, with an accuracy of 0.741, sensitivity of 0.692, specificity of 0.786, PPV of 0.750, NPV of 0.733, and AUC of 0.769, respectively. Although the performance of the LR model was not as good as the optimal model NB for the “Infarct_radiomics.” Overall, the model performance of “Hypoxic_radiomics” was better than that of the infarct regions. For example, for the SVM model, except for AUC, the performance of other parameters in the “Hypoxic_radiomics” was better than that in the “Infarct_radiomics.” Notably, the model performance of both infarct and hypoxic regions was significantly worse than that in the ischemic regions.

**Table 5:** Performance of perfusion radiomics features in infarct and hypoxic regions.

Classifier	Accuracy	Sensitivity	Specificity	PPV	NPV	AUC
SVM	0.704^1^/0.741^2^	0.692^1^/0.692^2^	0.714^1^/0.786^2^	0.692^1^/0.750^2^	0.714^1^/0.733^2^	0.830^1^/0.736^2^
MLP	0.704^1^/0.741^2^	0.769^1^/0.692^2^	0.643^1^/0.786^2^	0.667^1^/0.750^2^	0.750^1^/0.733^2^	0.736^1^/0.758^2^
RF	0.667^1^/0.704^2^	0.692^1^/0.615^2^	0.643^1^/0.786^2^	0.643^1^/0.727^2^	0.692^1^/0.688^2^	0.745^1^/0.747^2^
Ada	0.519^1^/0.741^2^	0.462^1^/0.615^2^	0.571^1^/0.857^2^	0.500^1^/0.800^2^	0.533^1^/0.706^2^	0.604^1^/0.709^2^
LR	0.704^1^/0.741^2^	0.692^1^/0.692^2^	0.714^1^/0.786^2^	0.692^1^/0.750^2^	0.714^1^/0.733^2^	0.846^1^/0.769^2^
NB	0.741^1^/0.704^2^	0.769^1^/0.692^2^	0.714^1^/0.714^2^	0.714^1^/0.692^2^	0.769^1^/0.714^2^	0.821^1^/0.736^2^

1: Infarct_radiomics and 2: Hypoxic_radiomics.

In addition, the perfusion radiomics features were combined from different regions to explore whether to improve the performance of models further. The performance of different combinations of perfusion radiomics features from ischemic, infarct, and hypoxic regions are provided in Table 6. In the “Ischemic_infarct_radiomics” group, the NB model performed well, with an accuracy of 0.815, sensitivity of 0.846, specificity of 0.786, PPV of 0.786, NPV of 0.846, and AUC of 0.849, respectively. The performance of this group was better than that of “Infarct_radiomics” and “Hypoxic_radiomics,” but inferior to “Ischemic_radiomics.” In the “Infarct_hypoxic_radiomics” group, the SVM model exhibited the best performance, with an accuracy of 0.741, sensitivity of 0.692, specificity of 0.786, PPV of 0.750, NPV of 0.733, and AUC of 0.863, respectively. This group was better than “Infarct_radiomics” overall and exhibited similar performance to “Hypoxic_radiomics” but worse than “Ischemic_radiomics” and “Ischemic_infarct_radiomics.” In the “Ischemic_hypoxic_radiomics” group, the RF model had the best performance, with accuracy of 0.852, sensitivity of 0.769, specificity of 0.929, PPV of 0.909, NPV of 0.813, and AUC of 0.934, respectively. This group overall outperformed “Infarct_radiomics” and “Hypoxic_radiomics,” as well as “Ischemic_infarct_radiomics” and “Infarct_hypoxic_radiomics,” but was still inferior to “Ischemic_radiomics.” In the “Ischemic_infarct_hypoxic_radiomics” group, the NB model had the best performance, with accuracy of 0.815, sensitivity of 0.846, specificity of 0.786, PPV of 0.786, NPV of 0.846, and AUC of 0.868, respectively. This group overall outperformed “Infarct_radiomics,” “Hypoxic_radiomics” and “Infarct_hypoxic_radiomics,” exhibited similar performance to “Ischemic_infarct_radiomics,” but was inferior to “Ischemic_hypoxic_radiomics” and notably inferior to “Ischemic_radiomics.”

**Table 6:** Performance of perfusion radiomics features in regional combinations.

Classifier	Accuracy	Sensitivity	Specificity	PPV	NPV	AUC
SVM	0.741^1^/0.741^2^/0.778^3^/0.741^4^	0.769^1^/0.692^2^/0.692^3^/0.692^4^	0.714^1^/0.786^2^/0.857^3^/0.786^4^	0.714^1^/0.750^2^/0.818^3^/0.750^4^	0.769^1^/0.733^2^/0.750^3^/0.733^4^	0.857^1^/0.863^2^/0.929^3^/0.863^4^
MLP	0.667^1^/0.667^2^/0.741^3^/0.815^4^	0.769^1^/0.692^2^/0.615^3^/0.846^4^	0.571^1^/0.643^2^/0.857^3^/0.786^4^	0.625^1^/0.643^2^/0.800^3^/0.786^4^	0.727^1^/0.692^2^/0.706^3^/0.846^4^	0.786^1^/0.824^2^/0.879^3^/0.846^4^
RF	0.704^1^/0.704^2^/0.852^3^/0.741^4^	0.692^1^/0.692^2^/0.769^3^/0.769^4^	0.714^1^/0.714^2^/0.929^3^/0.714^4^	0.692^1^/0.692^2^/0.909^3^/0.714^4^	0.714^1^/0.714^2^/0.813^3^/0.769^4^	0.838^1^/0.838^2^/0.934^3^/0.841^4^
Ada	0.704^1^/0.704^2^/0.852^3^/0.593^4^	0.615^1^/0.462^2^/0.846^3^/0.615^4^	0.786^1^/0.929^2^/0.857^3^/0.571^4^	0.727^1^/0.857^2^/0.846^3^/0.571^4^	0.688^1^/0.650^2^/0.857^3^/0.615^4^	0.764^1^/0.764^2^/0.868^3^/0.780^4^
LR	0.704^1^/0.741^2^/0.815^3^/0.667^4^	0.769^1^/0.692^2^/0.769^3^/0.692^4^	0.643^1^/0.786^2^/0.857^3^/0.643^4^	0.667^1^/0.750^2^/0.833^3^/0.643^4^	0.750^1^/0.733^2^/0.800^3^/0.692^4^	0.835^1^/0.852^2^/0.901^3^/0.808^4^
NB	0.815^1^/0.778^2^/0.815^3^/0.815^4^	0.846^1^/0.769^2^/0.769^3^/0.846^4^	0.786^1^/0.786^2^/0.857^3^/0.786^4^	0.786^1^/0.769^2^/0.833^3^/0.786^4^	0.846^1^/0.786^2^/0.800^3^/0.846^4^	0.849^1^/0.846^2^/0.923^3^/0.868^4^

1: Ischemic_infarct_radiomics, 2: Infarct_hypoxic_radiomics, 3: Ischemic_hypoxic_radiomics, and 4: Ischemic_infarct_hypoxic_radiomics.

This study evaluated the performance of original perfusion parameters to verify the superiority of radiomics features over original perfusion parameters in accurately assessing the degree of neurological impairment in AIS patients. Table 7 shows the performance of original perfusion parameters in different regions. In the “Ischemic_parameters” group, the MLP model exhibited the best performance, with an accuracy of 0.889, sensitivity of 0.846, specificity of 0.929, PPV of 0.917, NPV of 0.867, and AUC of 0.868, respectively. This group outperformed the perfusion radiomics feature groups of “Infarct_radiomics” and “Hypoxic_radiomics” and also outperformed the combinations of perfusion radiomics features from different regions but was substandard to “Ischemic_radiomics.” In the “Infarct_parameters” group, the best model SVM achieved an accuracy of 0.741, sensitivity of 0.769, specificity of 0.714, PPV of 0.714, NPV of 0.769, and AUC of 0.797, respectively. The overall model performance was consistent with the “Infarct_radiomics” but worse than “Ischemic_parameters.” In the “Hypoxic_parameters” group, the overall performance of the model was slightly lower than that of the “Ischemic_parameters” group but superior to that of the “Infarct_parameters” and the radiomics features groups “Infarct_radiomics” and “Hypoxic_radiomics.” The best model, RF, achieved an accuracy of 0.852, sensitivity of 0.769, specificity of 0.929, PPV of 0.909, NPV of 0.813, and AUC of 0.876, respectively.

**Table 7:** Performance of original perfusion parameters in ischemic, infarct, and hypoxic regions.

Classifier	Accuracy	Sensitivity	Specificity	PPV	NPV	AUC
SVM	0.778^1^/0.741^2^/0.667^3^	0.769^1^/0.769^2^/0.615^3^	0.786^1^/0.714^2^/0.714^3^	0.769^1^/0.714^2^/0.667^3^	0.786^1^/0.769^2^/0.667^3^	0.868^1^/0.797^2^/0.720^3^
MLP	0.889^1^/0.630^2^/0.667^3^	0.846^1^/0.615^2^/0.692^3^	0.929^1^/0.643^2^/0.643^3^	0.917^1^/0.615^2^/0.643^3^	0.867^1^/0.643^2^/0.692^3^	0.868^1^/0.736^2^/0.830^3^
RF	0.667^1^/0.741^2^/0.852^3^	0.692^1^/0.846^2^/0.769^3^	0.643^1^/0.643^2^/0.929^3^	0.643^1^/0.688^2^/0.909^3^	0.692^1^/0.818^2^/0.813^3^	0.788^1^/0.797^2^/0.876^3^
Ada	0.630^1^/0.741^2^/0.667^3^	0.692^1^/0.615^2^/0.769^3^	0.571^1^/0.857^2^/0.571^3^	0.600^1^/0.800^2^/0.625^3^	0.667^1^/0.706^2^/0.727^3^	0.720^1^/0.791^2^/0.753^3^
LR	0.778^1^/0.630^2^/0.667^3^	0.769^1^/0.692^2^/0.615^3^	0.786^1^/0.571^2^/0.714^3^	0.769^1^/0.600^2^/0.667^3^	0.786^1^/0.667^2^/0.667^3^	0.852^1^/0.786^2^/0.753^3^
NB	0.852^1^/0.704^2^/0.778^3^	0.769^1^/0.692^2^/0.692^3^	0.929^1^/0.714^2^/0.857^3^	0.909^1^/0.692^2^/0.818^3^	0.813^1^/0.714^2^/0.750^3^	0.863^1^/0.731^2^/0.863^3^

1: Ischemic_parameters, 2: Infarct_parameters, and 3: Hypoxic_parameters.

Moreover, the performance of the combined original perfusion parameters from different regions is also evaluated, as provided in Table 8. In the “Ischemic_infarct_parameters” group, the best model SVM achieved an accuracy of 0.704, sensitivity of 0.692, specificity of 0.714, PPV of 0.692, NPV of 0.714, and AUC of 0.857, respectively. This performance was far below that of the perfusion radiomics features groups and the uncombined original perfusion parameter groups. In the “Infarct_hypoxic_parameters” group, the best model RF achieved an accuracy of 0.778, a sensitivity of 0.846, a specificity of 0.714, a PPV of 0.733, NPV of 0.833, an AUC of 0.868, respectively, the performance was superior to “Infarct_parameters,” but inferior to that of “Ischemic_parameters” and “Hypoxic_parameters.” In the “Ischemic_hypoxic_parameters” group, the best model RF achieved an accuracy of 0.778, a sensitivity of 0.692, a specificity of 0.857, PPV of 0.818, NPV of 0.750, an AUC of 0.898, respectively, still inferior to “Ischemic_parameters” and “Hypoxic_parameters.” In the “Ischemic_infarct_hypoxic_parameters” group, the best model MLP achieved an accuracy of 0.741, the sensitivity of 0.846, specificity of 0.643, PPV of 0.688, NPV of 0.818, and AUC of 0.824, respectively, with slightly better performance than “Infarct_parameters,” but inferior to “Ischemic_parameters” and “Hypoxic_parameters.”

**Table 8:** Performance of original perfusion parameters in regional combinations.

Classifier	Accuracy	Sensitivity	Specificity	PPV	NPV	AUC
SVM	0.704^1^/0.667^2^/0.741^3^/0.667^4^	0.692^1^/0.692^2^/0.615^3^/0.692^4^	0.714^1^/0.643^2^/0.857^3^/0.643^4^	0.692^1^/0.643^2^/0.800^3^/0.643^4^	0.714^1^/0.692^2^/0.706^3^/0.692^4^	0.857^1^/0.841^2^/0.846^3^/0.846^4^
MLP	0.704^1^/0.667^2^/0.704^3^/0.741^4^	0.615^1^/0.692^2^/0.538^3^/0.846^4^	0.786^1^/0.643^2^/0.857^3^/0.643^4^	0.727^1^/0.643^2^/0.778^3^/0.688^4^	0.688^1^/0.692^2^/0.667^3^/0.818^4^	0.720^1^/0.758^2^/0.824^3^/0.824^4^
RF	0.667^1^/0.778^2^/0.778^3^/0.704^4^	0.692^1^/0.846^2^/0.692^3^/0.692^4^	0.643^1^/0.714^2^/0.857^3^/0.714^4^	0.643^1^/0.733^2^/0.818^3^/0.692^4^	0.692^1^/0.833^2^/0.750^3^/0.714^4^	0.808^1^/0.868^2^/0.898^3^/0.841^4^
Ada	0.704^1^/0.704^2^/0.704^3^/0.593^4^	0.462^1^/0.615^2^/0.615^3^/0.385^4^	0.929^1^/0.786^2^/0.786^3^/0.786^4^	0.857^1^/0.727^2^/0.727^3^/0.625^4^	0.650^1^/0.688^2^/0.688^3^/0.579^4^	0.758^1^/0.703^2^/0.742^3^/0.610^4^
LR	0.667^1^/0.667^2^/0.741^3^/0.667^4^	0.692^1^/0.769^2^/0.769^3^/0.692^4^	0.643^1^/0.571^2^/0.714^3^/0.643^4^	0.643^1^/0.625^2^/0.714^3^/0.643^4^	0.692^1^/0.727^2^/0.769^3^/0.692^4^	0.813^1^/0.852^2^/0.879^3^/0.835^4^
NB	0.704^1^/0.704^2^/0.778^3^/0.704^4^	0.692^1^/0.769^2^/0.692^3^/0.769^4^	0.714^1^/0.643^2^/0.857^3^/0.643^4^	0.692^1^/0.667^2^/0.818^3^/0.667^4^	0.714^1^/0.750^2^/0.750^3^/0.750^4^	0.769^1^/0.797^2^/0.885^3^/0.819^4^

1: Ischemic_infarct_parameters, 2: Infarct_hypoxic_parameters, 3: Ischemic_hypoxic_parameters, and 4: Ischemic_infarct_hypoxic_parameters.

**Figure 6:**
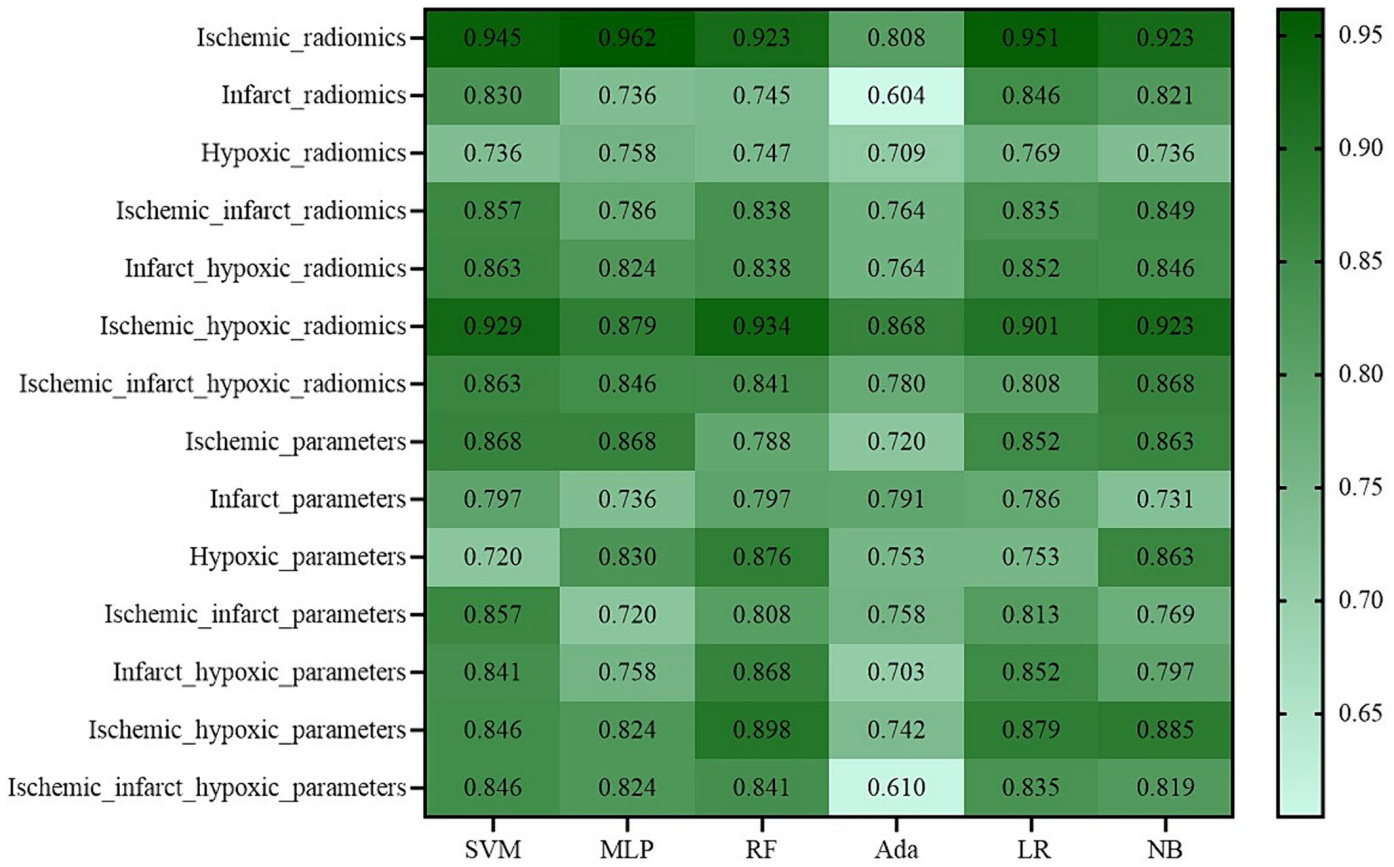
Heatmap of the performance (AUC) for all experimental groups.

To provide a clearer comparison of the performance across all experimental groups, Figure 5 shows the AUC values of the perfusion radiomics features and original perfusion parameters in experimental groups in the ischemic, infarct, and hypoxic regions. The graph shows that in the radiomics features experimental groups, the “Ischemic_radiomics” group exhibited the highest AUC values compared to the other experimental groups, followed by “Ischemic_hypoxic_radiomics.” Notably, in the RF and Ada models, the AUC values of the “Ischemic_hypoxic_radiomics” group even surpassed those of the “Ischemic_radiomics” group (0.934 vs. 0.923, 0.868 vs. 0.808). The performances of the remaining combinations of radiomics features groups were similar, although inferior to “Ischemic_radiomics” and “Ischemic_hypoxic_radiomics,” notably superior to “Infarct_radiomics” and “Hypoxic_radiomics.” Similarly, it is also observed the same pattern in the perfusion parameters experimental groups, where the “Ischemic_parameters” group performed the best, followed by “Ischemic_hypoxic_parameters.” The performances of the remaining combinations of perfusion parameters groups were similar and superior to the “Infarct_parameters” and “Hypoxic_parameters” groups. Furthermore, we found that within the same regional combination, the performances of the radiomics features groups surpassed that of the perfusion parameters groups. For instance, in the SVM model, the “Ischemic_radiomics” group improved by 7.7% compared with the “Ischemic_parameters” (0.945 vs. 0.868), “Infarct_radiomics” group improved by 3.3% compared with “Infarct_parameters” (0.830 vs. 0.797), and “Infarct_hypoxic_radiomics” group improved by 2.2% compared with “Infarct_hypoxic_parameters” (0.863 vs. 0.841). However, neither the combined perfusion radiomics features groups nor the combined original perfusion parameter groups showed performance improvement; both were inferior to “Ischemic_radiomics” and “Ischemic_parameters,” only showing slight improvement compared to the infarct and hypoxic regions. Additionally, the performance of the Ada model was notably inferior to other models (see Figure 6).

## 5 Discussion

Neurological impairment’s accurate assessment is crucial for treating and recovering AIS patients. Thus, the analysis of perfusion and SvO_2_ parameters in patients provides more insights into cerebral perfusion and oxygenation status, which enables physicians to assess the severity of the stroke and guide treatment selection and adjustment. In the past, many studies have demonstrated the value of perfusion and SvO_2_ parameters in stroke patients’ status assessment and prognosis prediction (13, 35, 36). However, these studies only focused on the connection between the parameters and the patients but ignored the deeper information within the parameters. In contrast, our research study intended to explore the role of perfusion radiomics features in assessing the degree of neurological impairment in AIS patients. Our results showed that the performance of perfusion radiomics features in the ischemic regions was optimal. The highest AUC reached 0.923, representing a 5.49% improvement over the original perfusion parameters of the ischemic regions (from 0.868 to 0.923). It demonstrates the superiority of ischemic perfusion radiomics features in assessing the degree of neurological impairment.

### 5.1 Ischemic perfusion radiomics features provide more information

DSC-PWI uses contrast agents as a medium to monitor the blood flow process and can truly reflect the hemodynamics of cerebral microcirculation. Quantitative analysis of DSC-PWI provides CBV parameters, which indicates the size of the ischemic core, CBF, reflecting neuronal activity in ischemic tissue; MTT, which indirectly reflects cerebral perfusion; and Tmax is commonly used to reflect the residual function of the penumbral zone (37). These perfusion parameters are often used in stroke diagnosis and postoperative evaluation by characterizing the cerebral tissue’s blood supply and transport capacity. However, solely relying on perfusion parameters is insufficient to characterize the degree of complex neurological impairment. For example, in the group of original perfusion parameters, the performance of the “Ischemic_parameters” group was the best, with an AUC of its best model of only 0.868. In contrast, in the perfusion radiomics features group, the performance of perfusion radiomics features in both the infarct and ischemic regions surpassed that of the original perfusion parameters in the same regions (AUC: 0.821 vs. 0.797, 0.923 vs. 0.868). In contrast, in the perfusion radiomics features group, the performance of perfusion radiomics features in both the infarct and ischemic regions surpassed that of the original perfusion parameters in the same regions. The perfusion radiomics features exhibited an AUC of 0.821 compared to 0.797 of original parameters in the infarct region and 0.923 compared to 0.868 in the ischemic region. It suggests that the radiomics features provide additional information leading to a more accurate assessment of neurological impairment. The original perfusion parameters only provide quantitative measurements of blood flow, blood volume, and other parameters of brain tissue, with limited informational elements. On the other hand, radiomics can quantify a wide range of features and play a vital role in capturing clinical information that is not easily seen by the naked eye. This is particularly important for clinical problems that are difficult to describe with simple visual features.

It is also observed that the perfusion radiomics features and the original perfusion parameters performance in the ischemic region were superior to those in the infarct region, regardless of the feature type (AUC: 0.923 vs. 0.821, 0.868 vs. 0.797). This phenomenon may be due to biological differences between ischemic and infarct regions. Infarct regions represent irreversible lesion tissue. Once an infarction occurs, the imaging manifestations tend to be consistent, with differences mainly in location and size. On the contrary, tissue in the ischemic regions, although affected by ischemia, has not yet died and retains some recovery capacity. The results from the combined experimental groups indicate that both the perfusion radiomics features and the original perfusion parameters experienced a decrease in model performance after the combination compared to the ischemic regions, only showing improvement compared to infarct and hypoxic regions. This also demonstrates that the tissue in the ischemic regions may contain more information, potentially aiding in the study of stroke progression.

### 5.2 Hypoxic perfusion radiomics features demonstrated poor performance

Compared to cerebral hemodynamic analysis, cerebral oxygen metabolism may more directly reflect the neural functional vitality of brain tissue. Previous imaging studies on cerebral oxygen metabolism have primarily relied on positron emission tomography (PET) and blood oxygen level-dependent (BOLD) techniques, both suffer from low spatial resolution, poor specificity, and susceptibility to interference (38–40). SWI is a high-resolution three-dimensional imaging method based on phase-enhanced gradient echo, highly sensitive to the presence of venous blood, hemorrhage, and iron deposition. Venous oxygenation information can be obtained by measuring the phase difference between local veins and surrounding tissues (15). Some studies have used SWI to generate QSM for non-invasive calculation of SvO_2_, and some studies have shown a significant correlation between SvO_2_ in low-perfusion regions and the degree of neurological impairment in AIS patients (41). Similarly, a single parameter of SvO_2_ cannot accurately reflect the degree of neurological impairment, considering the current lack of accurate and reliable SvO_2_ maps. Therefore, in this study, the calculation of SvO_2_ was used to identify hypoxic regions, and perfusion radiomics features were extracted from these hypoxic regions to assess the degree of neurological impairment. However, the results showed that the performance of perfusion radiomics features in hypoxic regions was not improved. Instead, it was inferior to the original perfusion parameters in hypoxic regions (AUC: 0.769 vs. 0.876) and even worse than the perfusion radiomics features in ischemic and infarct regions (AUC: 0.769 vs. 0.923 and 0.821, respectively). One possible reason could be the difference between imaging techniques: hypoxic regions were calculated based on QSM. Ischemic regions were calculated based on perfusion parameter maps, and hypoxic regions may not be reflected in perfusion parameter maps. Another reason could be the difference in scanning time between PWI and SWI. The time gap between the two scans may cause the expansion of ischemic regions or the migration of hypoxic regions. This can lead to some discrepancies between ischemic and hypoxic regions. As a result, the hypoxic regions in the perfusion parameter maps did not yet exhibit abnormalities during scanning.

## 6 Limitations and future research directions

The methods and materials of this study have certain limitations. Firstly, the study was based on a single-center, small sample size of subjects, and the generalization and robustness of this method have not been validated. Additionally, due to the small amount of patient data, it was difficult to perform multi-category assessments, posing challenges for clinical application. To address this issue, we will expand the dataset and collect multi-center data in future work. Secondly, due to limitations in the calculation method of SvO_2_, all included patients in the study had unilateral lesions, which could not apply to all AIS patients. And in the process of reconstructing QSM, we used high-pass filtered phase, which may affect the accuracy of QSM. Therefore, we will continue to optimize the calculation and image reconstruction methods to achieve accurate SvO_2_ calculations for the entire brain. To improve this, we will refine the calculation method to achieve SvO_2_ calculation for the entire brain region. Furthermore, this study used NIHSS as the gold standard to assess the degree of neurological impairment in AIS patients. Although this scale has been widely used and recognized clinically, it is susceptible to subjective influences from scoring personnel. This subjectivity may mislead the classification decisions of machine learning classifiers. We will seek more objective evaluation criteria to validate the method’s accuracy in the future. Finally, despite the poor performance of perfusion radiomics features in hypoxic regions, we still believe that SvO_2_ can provide more information, and we will continue to study the value of SvO_2_ in the diagnosis and treatment of AIS.

## 7 Conclusion

This study extracted radiomics features and perfusion parameters from perfusion parameter maps (CBF, CBV, MTT, and Tmax) of ischemic, infarct, and hypoxic regions. The aim was to assess the degree of neurological impairment in AIS patients. Additionally, this research explored the role of ischemic perfusion radiomics features in assessing neurological impairment. Experimental results demonstrate that the performance of perfusion radiomics features in the ischemic regions surpasses that of other perfusion radiomics feature groups and the original perfusion parameters groups. The AUC reached 0.923, representing a 5.49% improvement over the original perfusion parameters in the ischemic regions (from 0.868 to 0.923). This method can provide a more objective tool for assessing the degree of neurological impairment in clinical practice, facilitating the development of personalized treatment plans for patients to improve prognosis and rehabilitation.

## Data availability statement

The raw data supporting the conclusions of this article will be made available by the authors, without undue reservation.

## Ethics statement

The studies involving humans were approved by the Ethics Committee of Shanghai Fourth People’s Hospital affiliated with the Tongji University School of Medicine, China (Approval number: 20200066-01; Approval Date, 15 May 2020). The studies were conducted in accordance with the local legislation and institutional requirements. The ethics committee/institutional review board waived the requirement of written informed consent for participation from the participants or the participants’ legal guardians/next of kin due to the retrospective nature of the study.

## Author contributions

JL: Conceptualization, Data curation, Formal analysis, Investigation, Methodology, Software, Validation, Visualization, Writing – original draft, Writing – review & editing. MY: Formal analysis, Investigation, Validation, Writing – review & editing. YG: Methodology, Software, Validation, Writing – review & editing. YY: Formal analysis, Visualization, Writing – review & editing. FC: Software, Validation, Visualization, Writing – review & editing. JF: Formal analysis, Investigation, Visualization, Writing – review & editing. AZ: Data curation, Formal analysis, Writing – review & editing. HH: Formal analysis, Writing – review & editing. XZ: Data curation, Validation, Writing – review & editing. XM: Data curation, Visualization, Writing – review & editing. HY: Validation, Visualization, Writing – review & editing. AC: Data curation, Software, Validation, Writing – review & editing. GH: Investigation, Validation, Writing – review & editing. TH: Data curation, Formal analysis, Writing – review & editing. YL: Conceptualization, Data curation, Investigation, Writing – review & editing. YK: Conceptualization, Data curation, Funding acquisition, Project administration, Resources, Supervision, Writing – review & editing.
